# Axillary Management in Breast Cancer Patients Undergoing Upfront Surgery: Results from a Nationwide Survey on Behalf of the Clinical Oncology Breast Cancer Group (COBCG) and the Breast Cancer Study Group of the Italian Association of Radiotherapy and Clinical Oncology (AIRO)

**DOI:** 10.3390/curroncol30080542

**Published:** 2023-08-08

**Authors:** Fiorenza De Rose, Riccardo Ray Colciago, Sara Lucidi, Eliana La Rocca, Agnese Prisco, Elisabetta Bonzano, Bruno Meduri, Maria Carmen De Santis, Samantha Dicuonzo, Nadia Pasinetti, Isabella Palumbo, Icro Meattini, Pierfrancesco Franco

**Affiliations:** 1 Department of Radiation Oncology, Santa Chiara Hospital, 38122 Trento, Italy; sara.lucidi@apss.tn.it; 2 Clinical Oncology Breast Cancer Group (COBCG), 50134 Firenze, Italy; riccardoray.colciago@istitutotumori.mi.it (R.R.C.); eliana.larocca1@gmail.com (E.L.R.); agnese.prisco@asufc.sanita.fvg.it (A.P.); elisabettabonzano@gmail.com (E.B.); meduri.bruno@aou.mo.it (B.M.); mariacarmen.desantis@istitutotumori.mi.it (M.C.D.S.); nadia_pasinetti@yahoo.it (N.P.); icro.meattini@unifi.it (I.M.); 3 Department of Radiation Oncology, Fondazione IRCCS Istituto Nazionale dei Tumori, 20133 Milano, Italy; 4 Department of Radiation Oncology, Azienda Ospedaliero Universitaria Integrata, 37126 Verona, Italy; 5 Department of Radiation Oncology, University Hospital of Udine, ASUFC, 33100 Udine, Italy; 6 Radiation Oncology Department, Fondazione IRCCS Policlinico San Matteo and University of Pavia, 27100 Pavia, Italy; 7 Department of Radiation Oncology, University Hospital of Modena, 41125 Modena, Italy; 8 Division of Radiotherapy, IEO, European Institute of Oncology IRCCS, 20122 Milan, Italy; samantha.dicuonzo@ieo.it; 9 Breast Cancer Group, Italian Association of Radiotherapy and Clinical Oncology (AIRO), 20124 Milano, Italy; isabella.palumbo@unipg.it; 10 Radiation Oncology Department, ASST Valcamonica Esine, University of Brescia, 25121 Brescia, Italy; 11 Radiation Oncology Section, Department of Surgical and Biomedical Science, University of Perugia, Perugia General Hospital, 06156 Perugia, Italy; 12 Department of Experimental and Clinical Biomedical Sciences “M. Serio”, University of Florence, 50121 Florence, Italy; 13 Radiation Oncology Unit—Oncology Department, Azienda Ospedaliero Universitaria Careggi, 50134 Florence, Italy; 14 Department of Translational Medicine, University of Eastern Piedmont & Radiation Oncology Unit, AOU “Maggiore della Carita”, 28100 Novara, Italy

**Keywords:** breast cancer, sentinel lymph node biopsy (SLNB), axillary lymph node dissection (ALND), node-positive disease, regional node irradiation (RNI), multidisciplinary discussion

## Abstract

Background: We assessed the current practice concerning the axillary management of breast cancer (BC) patients undergoing upfront surgery among radiation oncologists (ROs) practising in Italy. Methods: An online survey via SurveyMonkey (including 21 questions) was distributed amongst ROs in Italy through personal contacts and the Italian Association for Radiotherapy and Clinical Oncology (AIRO) network from August to September 2022. We particularly focused on the emerging omission of axillary lymph node dissection (ALND) in the presence of 1–2 sentinel node-positive patients and the consequent change in the role of regional nodal irradiation (RNI). Results: A total of 101/195 (51% response rate) Italian Radiotherapy Cancer Care Centres answered the survey. With respect to patients with 1–2 sentinel node-positive, the relative proportion of respondents that offer patients ALND a) always, b) only in selected cases, and c) never was 37.6%, 60.4%, and 2.0%, respectively, with no significant geographical (North vs. Centre–South Italy; *p* = 0.92) or institutional (Academic vs. non-Academic; *p* = 0.49) differences. Radiation therapy indications varied widely in patients who did not undergo ALND. Among these, about a third of the respondents (17/56, 30.4%) stated that RNI was constantly performed. On the other hand, half of the respondents offered RNI in selected cases, stating that an unfavourable biologic tumour profile and extracapsular nodal extension were considered drivers of their decision. Conclusions: Results of the present survey show the variability of axillary management offered in clinical practice for BC patients undergoing conserving surgery upfront in Italy. Analysis of these attitudes may trigger the modification of some clinical approaches through multidisciplinary collaboration and create the background for future clinical investigations.

## 1. Introduction

Axillary management is crucial in breast cancer (BC) treatment. Historically, axillary lymph node dissection (ALND) was considered standard to complete staging and detect any potential lymph node metastasis [1].

However, ALND is associated with a high morbidity rate, significantly impacting patients’ health-related quality of life [2]. The introduction of the sentinel lymph node biopsy (SLNB), supported by the results of prospective randomised studies, has changed the management of early-stage BC with clinically negative axilla, with this technique becoming a standard approach [3]. At the same time, ALND continued to be recommended for most patients with clinically positive axillary nodes and/or positive SLNB.

The use of ALND after a positive SLNB was subsequently questioned, given that about half of the patients with macrometastatic sentinel lymph nodes (size > 2 mm) and even more with micrometastases (<2 mm) who underwent ALND had no other positive lymph nodes. Moreover, an extremely low number of axillary recurrences in patients with negative SLNB was observed (<1%) [4]. The introduction of more effective postoperative therapies (both loco-regional and systemic), the chance for tumour downstaging with neoadjuvant approaches, and a better understanding of tumour biology pushed the trend toward de-escalation of axillary surgery in this setting [5]. Different options for axillary management in patients with early BC and positive SLNB were investigated in randomised controlled trials.

The results of the ACOSOG-Z0011 study [6] showed non-inferiority in terms of loco-regional control, overall survival (OS), and disease-free survival (DFS) between the two randomised groups (axillary dissection vs. no dissection). Patients with T1–2 BC were included and offered breast-conserving surgery (BCS) with whole breast irradiation (WBI), provided that 1–2 positive sentinel nodes (micro or macroscopic disease) were found. Two other randomised trials, AMAROS [7,8] and OTOASOR [9], investigated the role of axillary irradiation as an alternative to ALND in patients with macrometastatic SLNB. After a median follow-up of 10 and 8 years, respectively, these studies did not report statistically significant differences in axillary disease relapse, OS, or DFS between axillary surgery and radiation. Moreover, in the AMAROS trial, a lower lymphedema rate was found in the radiotherapy (RT) arm.

The results of these studies led to a change in clinical practice [10], supported by international clinical guidelines such as those of ASCO and NCCN [11,12]. These studies recommend the omission of ALND in the presence of a positive sentinel lymph node (SNL) for macrometastases in cases of breast-conserving therapy, reserving regional nodal irradiation (RNI) for high-risk cases.

Nevertheless, European countries are currently slightly more conservative since no uniform attitude exist and clear recommendations still have to be defined. Thus, many studies have been performed, and mature data are awaited to corroborate the results of the ACOSOG-Z0011 study within a better methodological framework (SINODAR ONE in Italy, POSNOC in the UK, SENOMAC in Sweden, and BOOG 2013-07 in the Netherlands) [13,14,15,16].

In 2020, the Italian Senonetwork study group updated a document on axillary management to underline the importance of a multidisciplinary discussion in the case of 1–2 macrometastases after SLNB. The document emphasised the consideration of omitting ALND after breast-conserving therapy in patients that received whole breast irradiation, as reported in the AIRO Breast Cancer Group Best Clinical Practice [17]. A careful evaluation of risk factors (age, co-morbidities, and molecular subtypes) was recommended to properly define the therapeutic strategy and the role of regional nodal irradiation. Therefore, additional sub-analysis according to the biological characteristics of the disease is needed to establish the impact of ALND omission (or other adjuvant approaches) on unexpectedly positive SLNB [18].

These new surgical axillary approaches inevitably lead to a change in the role and magnitude of the clinical benefit of nodal RT. Specifically, with respect to the indication and extension of treatment volumes, these approaches result in an extremely heterogeneous clinical practice. This survey aimed to report the practice of Radiation Oncologists (ROs) working in Italy concerning the axillary management of BC and shed light on this highly debated topic.

## 2. Materials and Methods

An anonymous, voluntary survey-based questionnaire was administered to ROs practising in Italy through personal contacts and the Italian Association for Radiotherapy and Clinical Oncology (AIRO) network from August to September 2022. The survey was created with the SurveyMonkey platform [http://www.SurveyMonkey.com]. Specifically, it included 21 consecutive questions aimed at covering different items: demographics (6 items), expertise in BC treatment (3 items), surgical management (5 items), RNI (6 items), and dosimetric evaluation (1 item) (see File S1). We particularly focused on the emerging omission of ALND in the presence of 1–2 sentinel node-positives and the consequent change in the role of RNI.

Questions were designed on a multi-choice frame, allowing for multiple responses and free-text replies when required. The questionnaire items were defined over a multistep process by a panel of experts in the Clinical Oncology Breast Cancer Group (COBCG) and AIRO Breast Cancer study groups. No incentives were offered to provide the survey results. Adaptive questioning (only conditionally displayed based on responses to other items—questions 10 and 15) was used to reduce the complexity of the survey. In specific questions, more than one answer was allowed, and some were asked to rate the importance on a 5-point Likert scale. An IP address was used to define a unique visitor per centre (the most recent entries were kept for analysis, and the others were eliminated). The survey was conducted according to the CHERRIES statement [19] and was revised and approved by the scientific committee of AIRO (Nr. 15/2023).

Chi-squared testing was used to test for statistically significant differences in the management of the axilla. Participating ROs were geographically and institutionally allocated as North vs. Centre–South Italy and Academic vs. Non-Academic Hospital. The significance level was assessed if the *p*-value was ≤0.05.

## 3. Results

### 3.1. General Items: Demographics and Expertise in BC Treatment

A total of 101 out of 195 (51% response rate) Italian Radiotherapy Cancer Care professionals answered the survey. ROs with more than ten years of experience were 72 (71.3%). Eighty-five (83.3%) respondents worked in a public hospital, and more than half (61.5%) operated in non-academic hospitals. Most institutions (65%) treated more than 200 BC patients/year, with 48% of the respondents personally evaluating ≥100 cases/year; multidisciplinary boards discussed more than 75% of the cases for 85 (84.1%) of the respondents, as shown in Table 1. Three-dimensional conformal radiation therapy (3DCRT), intensity modulated radiation therapy (IMRT), and volumetric modulated arc therapy (VMAT) were the mainly employed radiation therapy techniques (94.1%, 88.2%, and 87.3%, respectively).

### 3.2. Surgical Management in Patients with Early Breast Cancer and 1–2 Macrometastases after BLS

**Figure 1:**
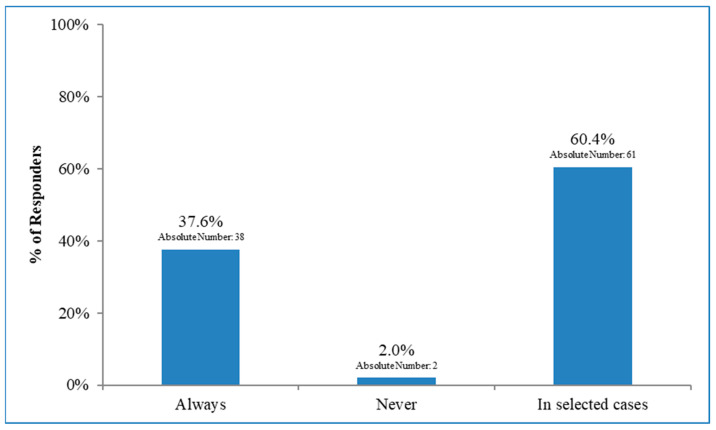
Frequency of axillary dissection for patients with 1–2 sentinel lymph node-positive.

**Figure 2:**
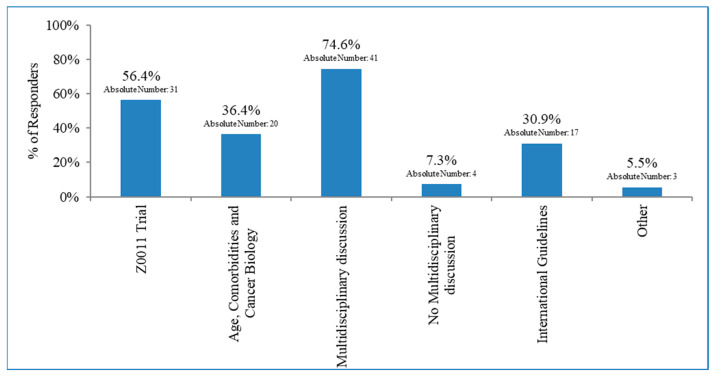
Factors mentioned in the survey as drivers of axillary surgery omission.

As shown in Figure 1, regarding patients with 1–2 sentinel node-positive, the proportion of respondents declaring that patients always, only in selected cases, and never undergo ALND was 37.6%, 60.4%, and 2.0%, respectively, with no significant geographical (North vs. Centre–South Italy; *p* = 0.92) or institutional (Academic vs. non-Academic; *p* = 0.49) differences. Referring to the period from 2019 to 2021, 29 (50.4%) of the respondents declared >50% of the patients not to be given an indication for ALND; only 15 (26.5%) physicians reported that surgery was not given in <25% of the cases. Moreover, 89.3% of the ROs stated that the number of patients having ALND omitted increased during the last three years (2019–2021). Excluding the centres where ALND is always performed, the reason for the omission of ALND was independent of the initial surgery in 59.7% of the cases (37/62), with 40.3% of the patients receiving breast-conservative surgery. In the 12th item (multiple choice), ROs indicated the driving factors for ALND omission: “discussion within a multidisciplinary board,” “Z0011 criteria” [6], and “patients’ age, clinical co-morbidities, and tumour biology” were indicated by 74.6%, 56.6%, and 36.4%, respectively. A subgroup of the answers (30.9%) affirmed that the breast surgeons decided according to international guidelines [11,12]. There was no multidisciplinary discussion in 7.3% of the cases. Only one RO stated that it was due to participation in a clinical trial for node-positive breast cancer patients (Figure 2).

### 3.3. Lymph Node Irradiation in Patients with Early Breast Cancer and 1–2 Macrometastases after BLS Who Did Not Undergo Axillary Dissection

**Figure 3:**
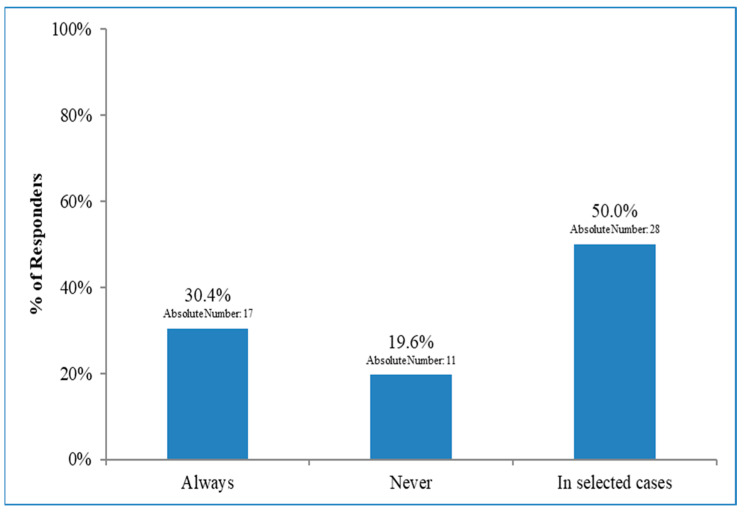
Regional nodal irradiation in patients who did not undergo axillary dissection.

**Figure 4:**
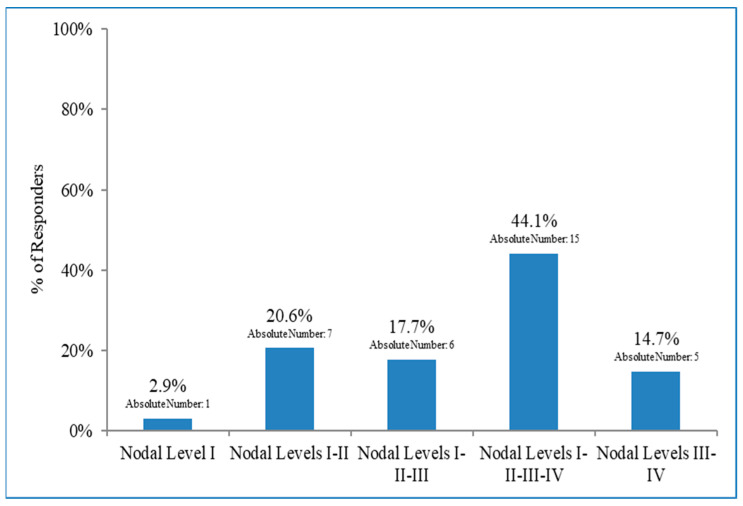
Target volume for regional nodal irradiation.

Radiation therapy indications varied widely among patients who did not undergo ALND. About a third of the respondents to the 15th item (17/56, 30.4%) stated that RNI was constantly performed. Conversely, 11/56 (19.6%) indicated that RNI was not prescribed. Half of the respondents offered RNI in selected cases (Figure 3), stating that unfavourable biologic tumour profiles and extracapsular nodal extension were considered drivers for their decision. The median score for both variables was 1 (fundamental criteria) (range 1–5). The total number of macrometastases, patient’s age, presence of vascular invasion, and tumour size were the driving criteria for 96.3%, 92.6%, 92.6%, and 85.2% of the ROs, respectively. The number of involved nodes and the patients’ age were considered fundamental criteria (median score 1, 1–5). Vascular invasion and tumour size were valued as essential criteria (median score 2, 1–5). Menopausal status and the patient’s preference were considered only in 70.4% of the cases, with a median score of 3 (1–5) and 4 (1–5), respectively. One respondent answered that a multigenic predictive test for systemic therapy (e.g., Oncotype DX) was considered. Another professional suggested the importance of other diagnostic exams (i.e., echography and positron emission tomoscintigraphy). Thirty-four respondents specified the target volume for RNI (Figure 4). Not even half (15/34, 44.1%) of the physicians indicated that all the nodal levels (i.e., I to IV) had to be treated. A minor part of the answers (6/34, 17.6%, and 7/34, 20.6%) suggested a treatment excluding the IV and III/IV levels, respectively. The proportion of ROs treating only the III and IV levels was 14.7%. The I level alone was the only volume in 3% of the cases. With respect to the treatment schedule, there was a substantial balance between moderate hypofractionation (40.05–42.40 Gy in 15–16 fractions) and normo-fractionated schedules (50 Gy in 25 fractions) (51.4% vs. 45.7% of the answerers, respectively). Only 1 (2.9%) RO prescribed both schedules depending on different factors. Prevalent irradiation techniques (94.3%) were IGRT/VMAT/helicoidal tomotherapy, but 3DCRT was still chosen by 31.4% of the respondents.

### 3.4. Dosimetric Evaluation

Twenty-seven physicians specified dose constraints for optimising the treatment plan, as reported in the supplementary material (Table S1). Table 2 summarises the constraints considered essential at least for 50% (14/27) of the respondents and their concordance. The ipsilateral lung and the heart were unanimously considered fundamental. Also, the contralateral breast (77.8%), the contralateral lung (66.7%), the left anterior descending coronary (66.7%), and the humeral head (55.6%) were indicated as organs at risk. In addition, the cervical esophagus, thyroid, brachial plexus, glottic larynx, and liver were suggested in 48.1%, 44.4%, 40.7%, 25.9%, and 22.2% of the cases, respectively.

## 4. Discussion

During the last decade, the regional lymph node management of patients with BC receiving upfront surgery has progressively changed. The ten-year updated results of the ACOSOG Z0011 trial [6] confirmed the safety of ALND omission in patients with microscopic and/or macroscopic metastases in up to two SLNBs who received WBI and adjuvant systemic therapy. Despite several limitations (unbalanced baseline characteristics—about 40% of participants had micrometastases), missing RT details, and a not negligible proportion of patients lost to follow-up, this study has laid the foundations for a significant change in surgical practice and had an immediate effect on clinical recommendations in the USA. Thus, the National Comprehensive Cancer Network (NCCN) and American Society of Clinical Oncology (ASCO) guidelines were modified, recommending no further axillary surgery in patients who meet the Z0011 selection criteria [11,12].

Conversely, European countries have yet to make explicit recommendations about axillary management in patients with 1–2 macrometastases after SLNB.

The results of our Italian survey confirmed an extremely heterogeneous scenario. Thus, most respondents (60.4%) declared that ALND was performed only in selected patients with 1–2 sentinel node-positives, whereas it was always performed in 37.6% of the centres. Moreover, 89.3% of the ROs stated an increase in the rate of ALND omission during the last three years (2019–2021). The Z0011 selection criteria and the multidisciplinary discussion were the main drivers for ALND’s omission. Still, patients’ age, clinical co-morbidities, and tumour biology also played a role in the choice of axillary management in more than one-third of the answers.

We are aware that this survey has some limitations. We recorded a relatively low response rate (51%), probably due to the short period available for survey compilation (45 days) and the mode of administration (voluntary e-mail survey). Thus, the present report may not be representative of all Italian centres. Despite that, according to the highest quality of care, 84% of the participating centres declared that a multidisciplinary discussion was performed in more than 75% of the cases. Moreover, most participating institutions (65%) treated more than 200 patients/year.

Necessarily, the omission of ALND influences the indications of postoperative treatments. Despite strong evidence supporting the significant impact of RNI on both breast cancer-specific and overall survival for most patients (data derived from newer trials reported by the EBCTCG meta-analysis preliminary results at SABCS2018), the nodal burden disease is currently still important to optimally tailor radiation therapy management.

RNI is still strongly indicated for patients reporting ≥4 positive axillary nodes [20] or 1–3 positive nodes and high-risk factors [21]. The Canadian MA.20 trial (including internal mammary, supraclavicular, and axillary nodal irradiation) and the EORTC 22922/10925 trial (including internal mammary and medial supraclavicular nodal irradiation) demonstrated the addition of RNI to improve DFS and BC mortality and recurrence, respectively, without showing benefit in OS. RNI advantage was found in patients with a relatively low regional disease burden even after receiving systemic therapy [22,23]. In all these trials, patients received RNI after ALND.

Conversely, the EORTC/AMAROS (After Mapping of the Axilla: Radiotherapy or Surgery?) trial enrolled patients with similar characteristics as Z011 to be randomised to completion of ALND or axillary RT [7]. After a median follow-up of 10 years, there were no significant differences in 10-year axillary recurrence, DMFS, or OS between the two groups; moreover, axillary irradiation was less morbid than ALND [8].

Both the surgical trend toward de-escalation of axillary management and the long-term results of the EORTC AMAROS trial call for a substantial redefinition of the role of RNI.

On this issue, our survey confirmed a wide variation in RNI among patients who did not undergo ALND, with a third of the respondents offering RNI to all patients and half of them offering RNI in selected cases. The driving criteria for offering RNI in selected cases included considerations of an unfavourable biologic profile, extracapsular nodal extension, number of macrometastases, age of the patients, vascular invasion, and tumour size.

Another debated topic was the extension of target volume when RNI was prescribed in patients with macrometastases who did not receive ALND. Most of the ROs stated that all the nodal levels (I to IV) had to be treated, but there is also no consensus in this field.

Finally, in the last decades, more patients have received RNI using highly conformal techniques (IMRT, VMAT, and helicoidal tomotherapy). This new attitude could reduce the “incidental dose” contribution in the nodal area not included in the target volume [24].

Moreover, albeit tumour biology and molecular subtype remain the milestones to manage and propose systemic therapy, new evidence supports the re-emerging role of nodal status for the decision-making process of adjuvant systemic therapies [25]. Indeed, in the MonarchE trial, patients with ≥4 positive nodes or 1 to 3 positive nodes and either tumour size ≥5 cm, histologic grade 3, or central Ki-67 ≥20% were randomised to receive adjuvant endocrine therapy (ET) with or without a CDK4/6 inhibitor (abemaciclib). The association (abemaciclib plus ET) significantly improved invasive Disease-Free Survival (iDFS).

Surely, recent advances in adjuvant treatments are leading the way to achieving better clinical outcomes for patients with early BC, but these data refer to a standard/old surgical approach. The introduction and wide diffusion of a de-escalation process should be tailored according to the risk of recurrence, trying to reduce the morbidity associated with therapies [26]. A joint effort of all actors involved in the multidisciplinary board is needed to identify the BC patients for whom it is possible to omit an axillary treatment safely. Additionally, it is essential to define what risk factors we have to consider for the intensification of surgical and adjuvant therapies [27].

## 5. Conclusions

Results of the present survey show the variability of axillary management offered in clinical practice for BC patients undergoing conserving surgery upfront. Analysis of these attitudes may give a chance to modify some clinical approaches through multidisciplinary collaboration and create the background for future clinical investigations in this debated field.

## Figures and Tables

**Table 1:** Respondents’ Characteristics.

Years of Experience	*<5*	15.8% (16) *	*5 ≤ x < 10*	12.9% (13)	*≥*10	71.3% (72)		
Geographical Distribution	*North*	47.5% (48)	*Centre*	19.8% (20)	*South*	32.7% (33)		
Institution of Work	*Academic Hospital*	42.5% (36)	*Non-Academic Hospital*	61.5% (62)	*Public Hospital*	83.3% (84)	*Private Hospital*	16.7% (17)
Irradiation Technique	*Brachy/IORT*	48.0% (49)	*3DCRT*	94.1% (96)	*IMRT/VMAT*	88.2% (90)	*Cyber/Tomo/* *Protons*	28.4% (29)
Pts/y Treated (within each Institution)	*<100*	8.0% (8)	*100 ≤ x < 200*	24.0% (24)	200 *≤ x* < 500	53% (53)	*≥*500	15.0% (15)
Pts/y Treated (by each Respondent)	*10 < x < 50*	16.0% (16)	*50 ≤ x < 100*	36.0% (36)	*≥*100	48% (48)		
Pts Discussed (with the MD Group)	*<25%*	2.0% (2)	*25% ≤ x < 50%*	1.0% (1)	50% *≤ x <* 75%	12.9% (13)	≥75%	84.1% (85)

Pts: patients; y: year; IORT: IntraOperative Radiation Therapy; 3DCRT: Three-Dimensional Conventional Radiation Therapy; IMRT: Intensity Modulated Radiation Therapy; VMAT: Volumetric Modulated Arch Therapy; MD: MultiDisciplinary. * Absolute numbers are reported in brackets.

**Table 2:** Organs at risk and dose constraints.

OARS	Dose Constraints	Concordance between Respondents
Ipsilateral Lung	V_20Gy_ < 15%	15% (4) *
	V_20Gy_ < 20%	19% (5)
	V_8Gy_ < 30–35%	15% (4)
	V_4Gy_ < 40–50%	19% (5)
Controlateral Breast	D_mean_ < 3 Gy	40% (11)
Heart	D_mean_ < 5 Gy	38% (10)
	D_mean_ < 4 Gy	26% (7)
LAD	D_mean_ < 20–25 Gy	55% (15)
	D_mean_ < 10 Gy	33% (9)
	V_40Gy_ < 1%	27% (8)
	V_30Gy_ < 2%	22% (6)
Humeral Head	D_mean_ < 40 Gy	27% (8)

OARS: Organs at Risk; V_20Gy_: Volume Receiving 20 Gy; V_8Gy_: Volume Receiving 8 Gy; V_4Gy_: Volume Receiving 4 Gy; D_mean_: Mean Dose; V_40Gy_: Volume Receiving 40 Gy; V_30Gy_: Volume Receiving 30 Gy; LAD: Left Anterior Descending Artery. * Absolute numbers are reported in brackets.

## Data Availability

The data presented in this study are available on request from the corresponding author.

## Supplementary Materials

The following supporting information can be downloaded at: https://www.mdpi.com/article/10.3390/curroncol30080542/s1, Table S1: Dose constraints for optimizing the treatment plan; File S1: List of Survey Questions and Items.
