# Supplementary material for: Axillary Management in Breast Cancer Patients Undergoing Upfront Surgery: Results from a Nationwide Survey on Behalf of the Clinical Oncology Breast Cancer Group (COBCG) and the Breast Cancer Study Group of the Italian Association of Radiotherapy and Clinical Oncology (AIRO)
Source: Curr Oncol. 2023 Aug 8;30(8):7489–98. doi: 10.3390/curroncol30080542 (PMC10453508; doi:10.3390/curroncol30080542)
Supplement: Supplementary file 1 [file curroncol-30-00542-s001.zip › curroncol-2475044-File S1.pdf]

Survey:

AXILLARY MANAGEMENT IN EARLY STAGE BREAST CANCER PATIENTS UNDERGOING UPFRONT SURGERY: PATTERN OF PRACTICE IN ITALY

Section 1: General items

Q1: Years of experience as a Radiation Oncologist

a) < 5 years
b) 5-10 years (5 to 10)
c) > 10 years

Q2: Italian region in which you practice your profession

a) Abruzzo
b) Basilicata
c) Calabria
d) Campania
e) Emilia-Romagna
f) Friuli-Venezia Giulia
g) Lazio
h) Liguria
i) Lombardia
j) Marche
k) Molise
l) Piemonte
m) Puglia
n) Sardegna
o) Sicilia
p) Toscana
q) Trentino-Alto Adige
r) Umbria
s) Valle d'Aosta
t) Veneto

Q3: Province in which you practice your profession

- Agrigento
- Alessandria
- Ancona
- Aosta
- Arezzo
- Ascoli Piceno
- Asti
- Avellino

- Bari
- Barletta-Andria-Trani
- Belluno
- Benevento
- Bergamo
- Biella
- Bologna
- Bolzano
- Brescia
- Brindisi
- Cagliari
- Caltanissetta
- Campobasso
- Carbonia-Iglesias
- Caserta
- Catania
- Catanzaro
- Chieti
- Como
- Cosenza
- Cremona
- Crotone
- Cuneo
- Enna
- Fermo
- Ferrara
- Firenze
- Foggia
- Forlì-Cesena
- Frosinone
- Genova
- Gorizia
- Grosseto
- Imperia
- Isernia
- La Spezia
- L'Aquila
- Latina
- Lecce

- Lecco
- Livorno
- Lodi
- Lucca
- Macerata
- Mantova
- Massa-Carrara
- Matera
- Messina
- Milano
- Modena
- Monza e Brianza
- Napoli
- Novara
- Nuoro
- Olbia-Tempio
- Oristano
- Padova
- Palermo
- Parma
- Pavia
- Perugia
- Pesaro e Urbino
- Pescara
- Piacenza
- Pisa
- Pistoia
- Pordenone
- Potenza
- Prato
- Ragusa
- Ravenna
- Reggio Calabria
- Reggio Emilia
- Rieti
- Rimini
- Roma
- Rovigo
- Salerno

- Medio Campidano
- Sassari
- Savona
- Siena
- Siracusa
- Sondrio
- Taranto
- Teramo
- Terni
- Torino
- Ogliastra
- Trapani
- Trento
- Treviso
- Trieste
- Udine
- Varese
- Venezia
- Verbano-Cusio-Ossola
- Vercelli
- Verona
- Vibo Valentia
- Vicenza
- Viterbo

Q4: Kind of Institution in which you currently work (multi-choice frame)

a) Highly specialized Hospital
b) Scientific Institute for Research and Healthcare
c) Academic Hospital
d) Non-academic Hospital
e) Residential care center

Q5: Kind of radiotherapy reimbursement systems in the Institution in which you work (multi-choice frame)

a) Public practice
b) Private practice
c) Agreed private practice

Q6: Radiation Therapy Techniques available in the Institute in which you currently work (multi-choice frame)

a) X-ray IORT
b) Electron beam IORT
c) Multi-catheter interstitial brachytherapy LDR
d) Multi-catheter interstitial brachytherapy HDR
e) Multi-catheter interstitial brachytherapy PDR
f) Balloon based brachytherapy
g) 3D-CRT
h) IMRT
i) VMAT
j) Tomotherapy
k) Cyberknife
l) Protontherapy
m) Other___________________

Q7: How many breast cancer patients are treated per year in the Radiation Oncology Department in which you currently work?

a) < 50
b) ≥50 e < 100
c) ≥100 e < 200
d) ≥200 e < 500
e) ≥500

Q8: How many breast cancer patients do you evaluate per year?

a) <10
b) ≥10 e < 50
c) ≥50 e < 100
d) ≥100

Q9: In your Institution, how many breast cancer patients (%) receive a treatment after a multidisciplinary discussion?

a) ≥ 75%
b) ≥ 50% e < 75%
c) ≥ 25% e < 50%
d) < 25%

Section 2: Surgical management in patients with early breast cancer and 1-2 macrometastases after BLS

Q10: In your Institution, is axillary dissection performed in patients with early-stage breast cancer after conservative surgery (BCS)/mastectomy and 1-2 macrometastases after sentinel lymph node biopsy (SLNB)?

a) Yes, always
b) No, never
c) In selected cases

If you answered "Yes, always", you finish the survey here

If you answered "No, never" or "in selected cases", you can continue the survey

Q11: In your Institution, what kind of surgery is performed in patients with early stage breast cancer and 1-2 macrometastases after BLS did not undergo axillary dissection

a) Breast Conservative Surgery (BCS)

b) Mastectomy

c) Both

Q12: If you answered "in selected cases" to the Q10 question, you can indicate which selection criteria are used in your Institution to omit axillary dissection in patients with with 1-2 macrometastases after SLNB (multi-choice frame)

a) Z0011 study criteria (T1-T2, with 1-2 macrometastases after SLNB, No neoadjuvant chemotherapy, Indication to Whole Breast Irradiation after breast conservative surgery)
b) Age, clinical comorbidities and tumor biology
c) Treatment is defined after discussion within a multidisciplinary board taking into account age, clinical comorbidities and tumor biology
d) There was no a multidisciplinary discussion
e) Breast surgeons decide according to international guidelines (ASCO, NCCN)
f) Other criteria (specify)

Q13: Over the last three years (2019-2021), how many patients with early stage breast cancer and 1-2 macrometastases after BLS did not undergo axillary dissection in your Institution?

a) ≥ 75%
b) ≥ 50% e < 75%
c) ≥ 25% e < 50%

d) < 25%

Q14: Over the last three years (2019-2021), have you detected an increased number of patients with early stage breast cancer and 1-2 macrometastases after BLS who did not undergo axillary dissection in your Institution?

a) Yes

b) No

Section 3: Lymph node irradiation in patients with early breast cancer and 1-2 macrometastases after BLS who did not undergo axillary dissection

Q15: In your Institution, in patients with early stage breast cancer and 1-2 macrometastases after BLS, who did not undergo axillary dissection, do you irradiate the breast only?

a) Yes, always
b) No, never
c) In selected cases

If you answered “Yes, always”, you finish the survey here

If you answered “No, never” or “in selected cases”, you can continue the survey

Q16: If you answered "In selected cases" to question Q15, please indicate which are the selection criteria that you evaluate to decide if regional nodal irradiation (RNI) is needed in your Institution?

Express your agreement about the selection criteria (to rate the importance) using a 5-point Likert scale.

Where required, specify your inclusion criteria (multi-choice frame)

a) Tumor size: □1 □2 □3 □4 □5

Inclusion criteria (specify):________________

b) Number of macrometastases after BLS: □1 □2 □3 □4 □5
   Inclusion criteria (specify):________________
c) Age: □1 □2 □3 □4 □5
   Inclusion criteria (specify):________________
d) Menopausal state: □1 □2 □3 □4 □5
e) Presence of Lymphovascular invasion (LVI): □1 □2 □3 □4 □5
f) Presence of extracapsular extension (ECE): □1 □2 □3 □4 □5
g) Unfavorable biologic profile: □1 □2 □3 □4 □5
   Inclusion criteria (specify):________________
h) Patient preference: □1 □2 □3 □4 □5
i) Other relevant factors (specify)________________________________

Q17: Usually, what was the "clinical target volume" in case of RNI?

a) Nodal level: 1
b) Nodal level: 1-2
c) Nodal level: 1-2-3
d) Nodal level: 1-2-3-4
e) Nodal level: 3-4

Q18: What Radiation Therapy Technique do you usually use for RNI? (multi-choice frame)

a) 3D-CRT
b) IMRT/VMAT/Tomotherapy
c) Protontherapy
d) Other (specify)_____________________

Q19: Which treatment schedule do you usually use for RNI?

a) Normo-fractionated scheme
b) Moderate hypofractionation
c) Other (specify)____________________

Q20: Indicate dose constraints (related to Whole Breast/RNI treatment plan) used for treatment plan optimization

a) Lung Dose Constraints ____
b) Heart Dose Constraints ____
c) LAD Dose Constraints ____
d) Brachial plexus Dose Constraints __________________________________
e) Humeral head Dose Constraints _____________________________________
f) Thyroid Dose Constraints ____

Section 4: Conclusion

Q21: Would you be joined in observational multicenter studies on lymph node irradiation in early breast cancers with 1-2 positive BLS for macrometastases did not undergo to axillary dissection?

a) Yes
b) No

If Yes, please specify your e-mail address:________________________________
