# Supplementary material for: Axillary Management in Breast Cancer Patients Undergoing Upfront Surgery: Results from a Nationwide Survey on Behalf of the Clinical Oncology Breast Cancer Group (COBCG) and the Breast Cancer Study Group of the Italian Association of Radiotherapy and Clinical Oncology (AIRO)
Source: Curr Oncol. 2023 Aug 8;30(8):7489–98. doi: 10.3390/curroncol30080542 (PMC10453508; doi:10.3390/curroncol30080542)
Supplement: Supplementary file 1 [file curroncol-30-00542-s001.zip › curroncol-2475044-Table S1.pdf]

CONSTRAINTS Ipsilateral Lung (centres 26/28)

V30	V25	V20	V18	V17	V16	V10	V8	V5	V4	MLD
<9% (1)	<5% (2)									7 Gy (1)
<10% (1)		<10% (2)			<10% (1)					9 Gy (3)
<15% (1)<br>V31		<15% (4)			<15% (2)					10 Gy (1)
< 30% (1)		<20% (5)	<20% (1)	<20% (1)	<20% (2)		<20%			12 Gy (1)
		<25% (1)		<35% (2)						13 Gy (1)
		<30% (2)								16 Gy (1)
							<30-35% (4)	<40% (2)	<40-50% (5)	
						<50% (1)	<40% (1)	<60% (1)		
								<65% (2)<br>[V4.8]		
								<85% (1)		

CONSTRAINTS Contralateral Breast (centres 20/28)

**Dmean**	Dmax	D1%	D2%	D50%	
<1.5 Gy (1)	<2.4 Gy (1)	<2.4 Gy(1)	10 Gy (1)	<4.9 Gy (1)	
<3 Gy (8)					
<4 Gy (2)					
< 5 Gy (2)					
Minimizzare la V10 (4)					

CONSTRAINTS Heart (centres 26/28)

**Dmean**	V5	V8	V10	V15	V16	V17	V18	V20	V25	V40
<3Gy (6)<br>[2.5Gy-<br>3.2Gy]		<10% (2)	<5% (1)	<4% (1)	<5% (3)	<10% (1)	<5% (2)	<5% (1)	<5% (2)	<3% (2)
<4 Gy (7)		<15% (1)		<30% (1)				<10% (1)	<10% (3)	<5% (1)

<5 Gy (10)		<25% (1)						<12.5% (2)		
<8Gy (1)		<30% (4)								
<25Gy?	<40%									

CONSTRAINTS Left Anterior Descending Coronary (centres 18/28)

Dmax	Dmean	V40-45	V30			
20 Gy (3)	20-25 Gy (7)	<1% (5)	<2% (4)			
30 Gy (1)	13 Gy (1)					
13 Gy (1)	10Gy (6)					

CONSTRAINTS Brachial Plexus (centres 10/28)

Dmax	Dmean	V60	D1%	
60 Gy (1)	54Gy (1)	< 5% (1)	<39.6 Gy (2)	
54 Gy ((2)				
52.5 Gy (1)				
50 Gy (1)				
45 Gy (1)				

CONSTRAINTS Humeral Head (centres 15/28)

Dmax	Dmean	V50	V45	D1%	D2%				
50 Gy (1)	40 Gy (4)	<10% (2)	< 5% (1)	<30 Gy (1)	<30 Gy (1)				
45 Gy (1)	35 Gy (1)		<10% (1)						
	25 Gy (1)								

CONSTRAINTS Cervical Oesophagus (centres 12/28)

Dmax	Dmean	V50	V40	V35	V28	D1%		
45 Gy (1)	<34 Gy (5)	<40% (2)	<40% (1)	<50% (4)	<45% (2)	<9-15 Gy (1)		
30 Gy (1)	<28 Gy (1)		<35% (1)					
15 Gy (1)	<25 Gy (1)							

	<9 Gy (1)							

CONSTRAINTS Glottic Larynx (centres 4/28)

Dmean	V50				
44-45Gy (2)	27% (2)				
30 Gy (1)					
25 Gy (1)					

CONSTRAINTS Liver (centres 4/28)

Dmean	V40	V15	V13		
<20 Gy (1)	<30% (1)	<10% (1)	<10% (1)		
<4 Gy (2)					

CONSTRAINTS Thyroid (centres 11/28)

Dmean	V45	V30	V16.1		
<30Gy (1)	<50% (3)	<62.5% (1)	<50% (2)		
<20 Gy (2)		<50% (1)			
<21 Gy (3)					

CONSTRAINTS Contralateral Lung (centres 16/28)

Dmean	V4	V5	V13.8	V20	
<8 Gy (3)	<10% (3)	<5% (1)	<3% (1)	<1% (1)	
<6 Gy (2)	<15% (1)	<10% (1)			
		<25% (V4.8) (1)			
<4 Gy (2)		<35% (1)			
<2.5Gy (1)		<46% (1)			
